# The Digital Pediatric Physiotherapy Framework (DPPF): A Systematic Review of Digital Health Integration in Pediatric Physiotherapy

**DOI:** 10.3390/children13040541

**Published:** 2026-04-13

**Authors:** Mshari Alghadier, Abdulmajeed S. Altheyab

**Affiliations:** 1 Department of Health and Rehabilitation Sciences, Prince Sattam bin Abdulaziz University, Alkharj 11942, Saudi Arabia; 2 Department of Occupational Therapy, College of Applied Medical Sciences, King Saud Bin Abdulaziz University for Health Sciences, Riyadh 11481, Saudi Arabia; althyaaba@ksau-hs.edu.sa; 3 King Abdullah International Medical Research Center, Riyadh 11481, Saudi Arabia

**Keywords:** pediatric physiotherapy, digital health, telerehabilitation, ICF-CY, implementation science

## Abstract

Background: Technology such as telerehabilitation, virtual reality, robotics, and wearable systems are reshaping pediatric physiotherapy. While evidence remains fragmented, there is little guidance on how these approaches can be integrated into coherent, family-centered care pathways. Objective: To develop the Digital Pediatric Physiotherapy Framework (DPPF) based on a systematic review of randomized evidence on digital interventions in pediatric physiotherapy. Methods: Several databases were searched for randomized trials published after 1 January 2020, including PubMed, Web of Science Core Collection, and Google Scholar. The included studies assessed the results of physiotherapist-delivered or physiotherapist-supervised digital interventions in children and adolescents aged 18 and younger. Population, intervention, outcome, implementation, and safety data were extracted. Considering the substantial heterogeneity of the findings, they were synthesized narratively. Cochrane RoB 2 was used to assess risk of bias, and GRADE was used to evaluate certainty of evidence. Results: Twenty-nine trials involving 1196 participants were included. Most studies examined virtual reality and gaming-based interventions, with fewer evaluating telerehabilitation/tele-exercise and robotic or wearable technologies. Digital interventions were most often directed at body-function and activity-level outcomes, while participation outcomes were less frequently studied. The strongest evidence supported short-term benefits in balance, gross motor function, upper-limb activity, pain, and selected fitness outcomes, particularly in children with cerebral palsy. Evidence for telerehabilitation and robotic or wearable approaches was more limited but generally promising. Implementation, equity, cost, and long-term outcomes were rarely reported. No eligible trial directly evaluated electronic patient-reported outcome measures, digital triage, or clinical decision support as stand-alone interventions. Conclusions: Digital interventions have the potential to strengthen pediatric physiotherapy, particularly for short-term motor and functional outcomes. The proposed DPPF provides an implementation-informed structure to guide future research, pathway design, and more purposeful integration of digital health into pediatric rehabilitation practice.

## 1. Introduction

Pediatric physiotherapy is undergoing rapid digital transformation, accelerated by the COVID-19 pandemic and advances in telehealth, wearable sensors, virtual reality (VR), and mobile applications [1,2,3,4,5]. These technologies may help improve access, engagement, and continuity of care for children with developmental, neurological, and musculoskeletal conditions, particularly when attendance at in-person services is affected by distance, transport, scheduling, or limited service availability [6]. They may also support remote assessment, home-based intervention, and follow-up. However, despite growing interest in these approaches, their integration into routine pediatric physiotherapy care remains variable, and there is still limited guidance on how best to incorporate them into structured clinical pathways [7,8,9].

In pediatric care, the rapid expansion of telehealth during the pandemic highlighted both the potential and limitations of digital rehabilitation. In many cases, remote delivery reduced travel burdens and improved flexibility, but it also presented challenges for technology access, caregiver confidence, and digital literacy, as well as difficulties conducting some assessments and treatment components remotely [10,11,12,13]. A significant portion of the literature has been devoted to specific technologies or specific populations, such as telerehabilitation, hybrid service models, digital assessment, wearable technology, and digital motor interventions. However, the focus has not been on how these tools may be integrated into the pediatric physiotherapy pathway in general [3,14,15,16,17,18]. There is thus no clear, implementation-informed framework for integrating these technologies into pediatric physiotherapy care as a result.

By integrating body functions, activities, participation, and contextual factors within a child- and family-centered model of care [19,20,21], the International Classification of Functioning, Disability, and Health for Children and Youth (ICF-CY) provides an important framework for pediatric rehabilitation. ICF-CY provides an important framework for framing clinical outcomes and goals; however, it does not fully address how digital interventions are introduced, adopted, and maintained in practice. Consequently, three implementation science frameworks were also used in this review. The Capability, Opportunity, Motivation-Behavior (COM-B) model was used to assess factors related to capability, opportunity, and motivation that may influence the adoption of digital approaches. The Reach, Effectiveness, Adoption, Implementation, Maintenance (RE-AIM) was included as a measure of extending the focus beyond effectiveness alone to include issues such as reach, adoption, implementation, and maintenance. As part of the Consolidated Framework for Implementation Research (CFIR), broader contextual influences were considered, including intervention characteristics, service settings, and implementation processes [22,23,24,25]. This review did not consider any single framework adequate, since digital pediatric physiotherapy involves behavioral uptake, service-level implementation, and broader contextual determinants not fully captured by the ICF-CY or any single implementation framework.

Rather than acting as alternatives to one another, these frameworks were used as complementary lenses. As a result, digital pediatric physiotherapy can be considered at several levels, including the child and the family, the clinician, the service context, and the wider implementation environment, when taken together. In pediatric physiotherapy, the use of hybrid delivery models, family involvement, and coordination across settings are often necessary components of digital care. This was considered particularly important. The use of these frameworks along with the ICF-CY, therefore, provided a broader basis for understanding not only whether digital interventions are beneficial, but also how they may be implemented in practice.

Therefore, this study addressed the lack of a model for integrating digital health into pediatric physiotherapy that is clear and implement-informed. As part of the development of the Digital Pediatric Physiotherapy Framework (DPPF), we systematically reviewed recent randomized clinical trials (RCTs) involving digital interventions in pediatric physiotherapy, summarized their effectiveness and safety, and used those findings to inform our development. A combination of evidence synthesis, the ICF-CY, and selected implementation science frameworks was used in this review to provide a foundation for future research and practice that is more clinically relevant.

## 2. Materials and Methods

### 2.1. Review Design and Registration

This systematic review followed PRISMA-2020 guidelines [26], and was prospectively registered in PROSPERO (CRD420261309333).

### 2.2. Search Strategy and Study Selection

For the purpose of identifying studies published from January 2020 forward, a systematic literature search was conducted on 30 December 2025 in PubMed, Web of Science Core Collection, and Google Scholar. Our search strategy was centered around three main concepts: pediatric populations, physiotherapy and rehabilitation, and digital health. In this review, physiotherapy and rehabilitation are defined broadly as interventions delivered in these settings to improve movement, functional ability, physical performance, or participation. Similarly, motor-based interventions were defined broadly, including exercises, task-oriented practice, movement training, or related rehabilitation approaches aimed at improving motor function or performance, including gait, balance, posture, strength, coordination, upper limb use, functional mobility, and physical activity. The goal of this broad framing was to ensure that relevant digital interventions in pediatric physiotherapy were captured during the search and study selection processes.

As an example, the PubMed search strategy integrated free-text and controlled vocabulary via Boolean operators: (“child” [Title/Abstract] OR “children” [Title/Abstract] OR “adolescent” [Title/Abstract] OR “pediatric” [Title/Abstract] OR “paediatric” [Title/Abstract]) AND (“physiotherapy” [Title/Abstract] OR “physical therapy” [Title/Abstract] OR “rehabilitation” [Title/Abstract] OR “motor intervention” [Title/Abstract] OR “motor training” [Title/Abstract] OR “exercise therapy” [Title/Abstract]) AND (“digital health” [Title/Abstract] OR “telehealth” [Title/Abstract] OR “telerehabilitation” [Title/Abstract] OR “mobile health” [Title/Abstract] OR “mHealth” [Title/Abstract] OR “virtual reality” [Title/Abstract] OR “wearable” [Title/Abstract] OR “serious game” [Title/Abstract] OR “artificial intelligence” [Title/Abstract]). Equivalent adaptations were applied across other databases.

All references retrieved were exported to Mendeley Reference Manager (Elsevier, v.2.141.2). Duplicates were removed using Mendeley’s “Check for Duplicates” function, followed by manual verification. Titles and abstracts were screened by the authors (MG and AT) within Mendeley, and full texts of potentially eligible studies were then assessed for inclusion. Inclusion and exclusion decisions were reviewed by the authors (MG and AT), and any disagreements were resolved through discussion.

### 2.3. Eligibility Criteria

Randomized trials enrolling participants ≤18 years were included if they evaluated a physiotherapy or motor-rehabilitation intervention delivered or augmented by a digital modality such as VR, active videogaming, wearable robotics or sensors, tele-rehabilitation, exergaming without a head-mounted display, or any hybrid format and compared it with usual care, another digital modality, therapist-led treatment or a wait-list/attention control. Studies had to report at least one quantitative clinical outcome that could be mapped to an ICF-CY domain (body functions/structures, activities or participation). Publications in languages other than English, adult-only samples, non-controlled designs and conference abstracts were excluded.

### 2.4. Data Extraction

The two authors developed and piloted a Microsoft Excel workbook to ensure consistent data extraction across studies. The workbook contained predefined fields with data-validation drop-down menus for bibliographic details, country, population, sample size and age, intervention description (TIDieR-lite [27,28]), comparator, outcome measures and timing, adverse events, ICF-CY domains and use of implementation frameworks. Initial data extraction was completed by the first author (MG), and all entries were subsequently reviewed by the second author (AT). Any discrepancies were resolved through discussion and agreement between the two authors. In addition to clinical outcomes, information was also collected on the timing and delivery context of each intervention. These data were then synthesized to position the digital interventions within a standardized six-stage pediatric physiotherapy care pathway, spanning referral to long-term follow-up.

### 2.5. Risk-of-Bias Assessment and Data Synthesis

All randomized trials were appraised using the Cochrane RoB 2 tool [29]. Risk-of-bias judgments were cross-checked by both authors (MG and AT), and any differences were resolved through discussion until agreement was reached. Given the considerable heterogeneity across populations, interventions, comparators, and outcome measures, meta-analysis was not considered appropriate. Therefore, the findings were synthesized narratively, with structured tables used to summarize study characteristics, risk of bias, and the direction of findings.

To improve transparency in how intervention effects were interpreted, we applied predefined rules when summarizing study findings. Wherever possible, classification was based on the between-group result for the primary outcome identified by the original study. If no primary outcome was explicitly stated, the main outcome was determined from the stated study aim and the principal target of the intervention. When more than one measure was reported within the same outcome domain, all relevant measures were considered, but greater weight was given to the primary or main outcome and to the overall pattern of between-group findings across assessment time points. A study was classified as favoring the digital intervention when the main between-group comparison showed a statistically significant benefit for the digital intervention, as showing no between-group difference when no statistically significant between-group effect was found for the main outcome, and as mixed or control-favored when findings were inconsistent across outcomes or time points, or when the comparator performed better.

The majority of findings were integrated through a theory-informed narrative synthesis since only a few outcomes were sufficiently similar for quantitative comparison. By following best-practice guidelines [30], we systematically searched, extracted data from Excel, and developed a conceptual model of digital pediatric physiotherapy iteratively. In order to synthesize the findings from the included trials, a convergent approach was used, in which the findings were mapped onto a typical pediatric physiotherapy episode’s timeline. By linking digital functions to various phases of the clinical pathway, we identified remote triage, asynchronous assessment, and telemonitoring. By organizing digital technologies based on their clinical role rather than their technical format, the resulting six-stage care pathway represents the structural outcome of the evidence synthesis.

### 2.6. Certainty of Evidence

As part of our assessment of the certainty of evidence, we used the GRADE approach [31]. We considered five standard domains: bias risk, inconsistency, indirectness, imprecision, and publication bias. GRADE was applied at the outcome level rather than across the entire review due to the heterogeneity of populations, interventions, and outcome measures within the included studies. Due to the lack of quantitative pooling, overall certainty ratings were determined using a narrative assessment, which took into consideration factors such as consistency in finding directions across studies, overlap in confidence intervals reported, and the amount of evidence available.

### 2.7. Analytical Approach to Framework Development

Iterative deductive–inductive approaches were used to develop the DPPF. To provide a structure for organizing the evidence and defining the main domains of the model, established conceptual frameworks, including the ICF-CY and the implementation science frameworks COM-B, RE-AIM, and CFIR, were used. Despite offering a biopsychosocial and implementation-focused foundation, these frameworks were not rigidly applied. They were instead used as guiding lenses during analysis. Moreover, clinically relevant features emerged from the included studies and pathway mapping within pediatric physiotherapy care were incorporated via an inductive process. By doing so, the framework could reflect aspects that are particularly relevant to pediatric practice, including family involvement, developmental change, and the need for follow-up across different care stages. Physiotherapy was delivered through an iterative process of refining the framework until the identified digital functions matched both theoretical constructs and practical flow.

It was determined during synthesis that digital interventions were categorized by their clinical role within care rather than their technology type. Telerehabilitation, wearable sensors, and VR were mapped according to the functions they perform within the care pathway, such as communication, assessment, monitoring, treatment delivery, feedback, and follow-up. Rather than categorizing interventions using more than one digital modality separately, they were coded as integrated elements within the same pathway. As a result of this functional approach, the six-stage framework was developed and each digital modality was positioned according to the way it contributes to clinical care.

### 2.8. Ethics Statement

Ethical approval was not required for this study because it involved analysis of data from previously published studies only and did not include direct contact with human participants or access to identifiable personal data.

## 3. Results

### 3.1. Search Results

**Figure 1:**
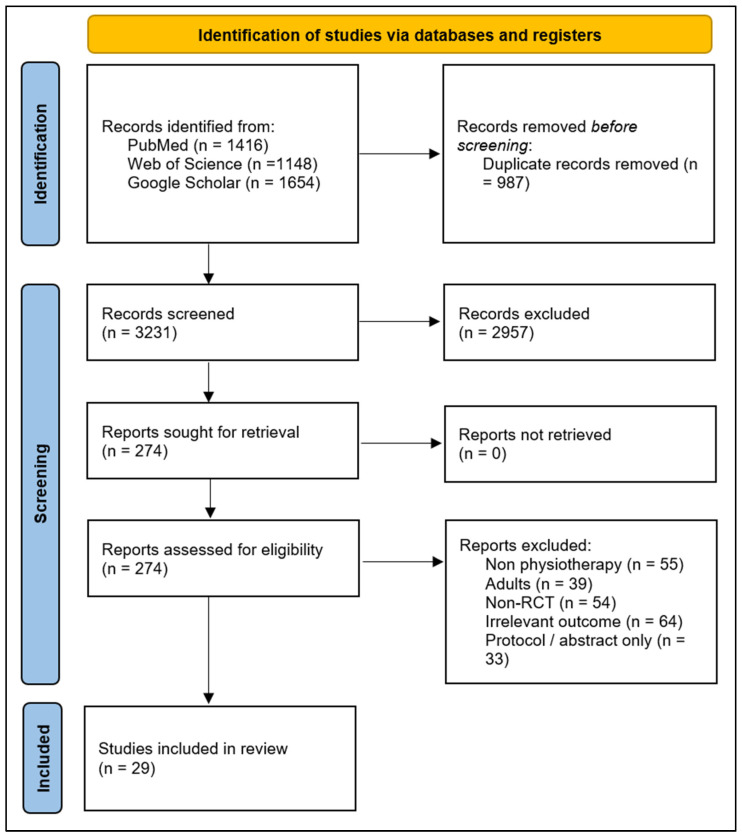
PRISMA 2020 flow diagram illustrating study selection for the systematic review of digital-rehabilitation interventions in pediatric populations.

The database search identified 4218 records. After removal of 987 duplicates, 3231 titles and abstracts were screened. 274 full-text articles were assessed for eligibility, and 29 randomized trials met the inclusion criteria and were included in the review (Figure 1).

### 3.2. Characteristics of Included Studies

A total of 29 trials (2020 to 2026) included in the review, involving 1196 participants. Sample sizes ranged from 10 to 148 participants per study. The included studies were RCTs, including 19 RCTs, three pilot RCTs, one feasibility RCT, one pragmatic RCT, one non-inferiority RCT, two cluster RCTs, one comparative RCT, and one crossover trial. Two studies were assigned to 2026 journal issues but fell within the review timeframe as online-ahead-of-print publications. The included studies covered a broad range of pediatric populations. Cerebral palsy (CP) was the most frequently studied condition, represented in 13 trials. Other populations included burn rehabilitation (5 trials), developmental coordination disorder (DCD) (2 trials), and autism spectrum disorder (ASD) (2 trials). The remaining studies included children or adolescents with juvenile rheumatoid arthritis, Duchenne/Becker muscular dystrophy, cystic fibrosis, obesity, and healthy adolescent or preadolescent populations, as well as one mixed-age rehabilitation sample that included pediatric participants. Individual study characteristics are summarized in Table 1.

### 3.3. Characteristics of Digital Interventions and Outcome Focus

Most included studies evaluated VR-, exergaming-, or video game-based interventions, which accounted for 22 of the 29 trials. These included immersive and non-immersive VR, Kinect-based interventions, Wii-based training, active videogames, and VR-enhanced treadmill or task-oriented training. Robotic or wearable technologies were evaluated in four studies, while telehealth-based interventions were examined in three studies. Comparators were usually active rehabilitation conditions, such as usual physiotherapy, conventional rehabilitation, gait training, task-specific exercise, or matched exercise-based programs. Fewer studies used wait-list, school-only, or sedentary control conditions. Overall, most trials evaluated digital approaches as an adjunct or alternative to established rehabilitation rather than against no treatment.

Across the included studies, interventions most often targeted activity and body-function domains of the ICF-CY, while participation-focused interventions were less common. Primary outcomes reflected the clinical aims of the individual studies and commonly included balance, gross motor function, gait speed, upper-limb function, range of motion, pain, exercise capacity, and quality of life. Broader participation outcomes and longer-term functional outcomes were evaluated less frequently.

### 3.4. Risk of Bias

**Figure 2:**
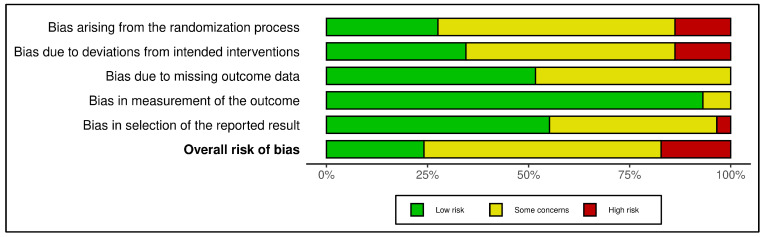
Summary of risk of bias judgments for the included studies using the Cochrane Risk of Bias 2 (RoB 2) tool. The bar chart shows the proportion of studies rated as low risk, some concerns, or high risk of bias across the five RoB 2 domains: bias arising from the randomization process, bias due to deviations from intended interventions, bias due to missing outcome data, bias in measurement of the outcome, and bias in selection of the reported result, as well as the overall risk of bias.

Risk of bias across the included trials was mixed. Of the 29 studies, 7 (24%) were judged to have an overall low risk of bias, 17 (59%) had some concerns, and 5 (17%) were judged to be at high risk of bias. Concerns were most commonly identified in the domains related to the randomization process and deviations from intended interventions, whereas the measurement of the outcome domain was rated as low risk in 27 of the 29 studies. Some concerns were also present in the domain relating to selection of the reported result. Overall, these findings suggest that the evidence base was more often limited by methodological uncertainty or reporting concerns than by consistently serious bias. The domain-level RoB 2 summary indicated that most studies were judged as having either low risk of bias or some concerns across domains, whereas high risk of bias was less frequent and was observed mainly in the randomization process, deviations from intended interventions, selection of the reported result, and in the overall risk of bias judgment (Figure 2). A traffic-light plot is provided in Supplementary Figure S1.

### 3.5. Quantitative Synthesis

Because of marked heterogeneity in populations, interventions, comparators, and outcome measures, a formal meta-analysis was not performed. Quantitative findings were therefore synthesized narratively using the predefined classification approach described in the Methods.

Overall, digital interventions more often showed benefit than no difference, although the direction and consistency of effects varied across conditions and outcome domains. The clearest pattern was seen in studies of VR-based interventions, particularly in children with CP, where improvements were most commonly reported for balance, gross motor function, gait-related performance, and upper-limb activity [36,37,44,45,46,49,56,59]. Pediatric burn rehabilitation had positive results as well [35,38,41,42,58], especially regarding pain, range of motion, and hand function. However, there was limited evidence regarding participation-level outcomes, longer-term follow-up, and integration into daily activities [33,47,53,54]. As a whole, the quantitative evidence suggests that digital interventions may improve selected motor and body function outcomes [36,37,41,44,45,46,49]. There is, however, a lack of consistency in conclusions across studies and populations, as well as heterogeneity and variable study quality [22,23,24,47,50,53,54].

### 3.6. Implementation and Safety Findings

Implementation-related outcomes were reported in several included trials, particularly those evaluating home-based VR, telehealth, and wearable robotics [39,47,50,54]. Feasibility, adherence, session completion, or fidelity were explicitly described in the home-based VR trial in children with CP, the telehealth ASD trial, the wearable-robot gait trial, and the tele-exercise trial in cystic fibrosis [39,47,50,54]. Parent or caregiver feedback, where collected, was generally positive, with families describing the interventions as acceptable and, in some cases, motivating [47,54]. Farr et al. (2021) [39] reported no adverse events in the home-based VR trial, Choi et al. (2024) [50] reported no adverse effects such as pain, skin lesions, increased fatigue, or falls during robot-assisted gait training, and Kilic et al. (2024) [54] found similar adverse-effect profiles across tele-exercise groups. Overall, no serious or lasting adverse events were clearly reported in these implementation-focused trials [39,50,54].

### 3.7. Certainty of Evidence Findings

Using the GRADE approach, the certainty of evidence for immersive or semi-immersive VR aimed at improving balance in children with CP was judged to be moderate, with the evidence downgraded by one level because of imprecision related to small sample sizes. For the other modality–outcome comparisons, including non-immersive VR or active videogames for gross motor and mobility outcomes, robot-assisted or wearable gait interventions, digital interventions in burn rehabilitation, upper-limb digital training, telerehabilitation/tele-exercise, and digital exercise interventions in mixed pediatric populations, the certainty of evidence was rated as low to very low. These lower ratings mainly reflected concerns related to risk of bias, inconsistency between studies, and imprecision in the reported estimates. Full GRADE assessments are presented in Table 2.

### 3.8. Summary of Evidence by Modality

#### 3.8.1. Telerehabilitation

Telerehabilitation was evaluated in a small number of trials, but the available evidence suggests that it is feasible and can produce outcomes comparable to face-to-face care in selected pediatric populations. In children with ASD, Su et al. (2023) found that tele-delivered fine-motor and social-skills training achieved treatment fidelity and developmental gains similar to face-to-face delivery [47]. In rural youth with obesity, Davis et al. (2024) reported favorable BMI-z outcomes with a tele-lifestyle intervention compared with a school-only program [53]. In children with cystic fibrosis, Kilic et al. (2024) found that home-based tele-exercise produced similar improvements in exercise-related outcomes to center-based care [54]. Taken together, these studies suggest that telerehabilitation may offer a practical alternative to in-person delivery in some contexts, although the evidence remains limited in scope and outcome diversity.

#### 3.8.2. Virtual Reality and Gaming Interventions

Virtual reality and gaming-based approaches were the most frequently studied digital modalities. In children with CP, several trials reported favorable effects on balance, gross motor performance, gait-related outcomes, and upper-limb function [36,37,44,45,46,49,56,59]. However, the magnitude and consistency of benefit varied across outcomes and study designs, and some studies reported non-inferiority rather than clear superiority [46]. Outside CP, VR-based interventions also showed promising findings in DCD, ASD, burn rehabilitation, Duchenne/Becker muscular dystrophy, juvenile rheumatoid arthritis, and selected adolescent or sports-related populations [32,33,41,42,48,51,55,57,60]. Overall, the evidence suggests that VR-based interventions may support short-term improvements in motor, pain-related, and selected functional outcomes, but the studies were heterogeneous in population, intervention dose, and primary endpoints.

#### 3.8.3. Wearable Sensors, Robotics, and Remote Monitoring

Evidence for robotics and wearable technologies was more limited than for VR, but several studies reported favorable findings. In children with CP, robotic-assisted gait and wearable exoskeleton interventions showed potential benefits for selected gait and motor outcomes, although results were not uniform across all measures [34,43,50]. A robot-assisted upper-limb program in children with hand burns also showed improvements in range of motion and grasp-related outcomes compared with conventional therapy [35]. In addition, motion-capture gaming and wearable activity monitoring were used to quantify treatment intensity or motor change in pragmatic and condition-specific trials [40,52]. Taken together, these findings suggest that robotics and sensor-based approaches may support treatment delivery and objective monitoring, but the evidence base remains smaller and generally less mature than that for VR-based interventions.

#### 3.8.4. Evidence Gaps in Digital Monitoring and Decision Support

No eligible trial evaluated electronic patient-reported outcome measures (e-PROMs), digital triage, or clinical decision support systems as stand-alone interventions. Some studies incorporated elements relevant to these functions, such as digitally captured patient-reported outcomes, treatment-fidelity checklists, or structured remote pathways [38,42,47,53] but these components were not evaluated as primary digital interventions. These areas should therefore be interpreted as evidence gaps rather than established modalities within the current review.

To complement the study-level and certainty-of-evidence summaries presented in Table 1 and Table 2, Table 3 provides a concise overview of the main digital health modalities identified in this review, their clinical role in pediatric physiotherapy, and the principal strengths, limitations, and evidence gaps relevant to practice and implementation.

## 4. Discussion

This review synthesized randomized evidence on digital health interventions in pediatric physiotherapy and used those findings to inform the DPPF. Overall, the included trials suggest that digital interventions may support short-term improvements in body-function and activity-level outcomes, particularly for balance, gross motor performance, gait-related outcomes, upper-limb function, pain, and selected fitness outcomes, with the strongest body of evidence centered on VR- and gaming-based interventions [36,37,41,44,45,46,49,56,59]. More limited but encouraging evidence was also identified for telerehabilitation, robot-assisted training, and wearable or sensor-supported approaches [34,35,39,43,47,50,53,54]. At the same time, the evidence base remains uneven. Most studies focused on the delivery of treatment, follow-up was generally short, and outcomes related to participation, maintenance, equity, and implementation at scale were infrequently reported [23,24,25,36,37,47,53,54].

A central implication of the review is that the available evidence supports some parts of the proposed framework more directly than others. The included trials provide the clearest support for the intervention-delivery stage of care, and to a lesser extent for selected aspects of monitoring and remote follow-up [39,40,47,50,54]. In contrast, digital triage, e-PROMs as stand-alone tools, clinical decision support, and digitally supported transitions were not directly evaluated as primary interventions in the included trials. These components should therefore be interpreted as framework-informed pathway elements, grounded in pediatric rehabilitation, implementation science, and adjacent digital health literature, rather than as areas already supported by equivalent levels of trial evidence [2,19,20,21,22,23,24,25,61,62,63]. This distinction is important because it preserves alignment between the empirical findings of the review and the broader conceptual contribution of the framework.

### 4.1. The Digital Pediatric Physiotherapy Framework (DPPF)

**Figure 3:**
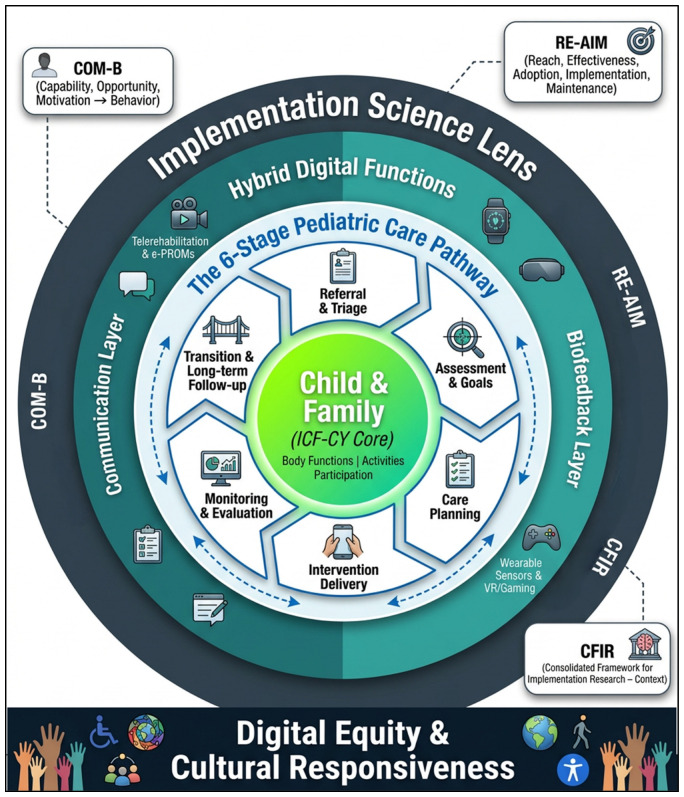
Digital Pediatric Physiotherapy Framework. The framework places the child and family at its center, with the ICF-CY providing the core structure for goals and outcomes across body functions, activities, and participation. Around this core is a six-stage pediatric physiotherapy care pathway, spanning referral, assessment, care planning, intervention delivery, monitoring, and transition/long-term follow-up. A surrounding ring of hybrid digital functions includes a communication layer (e.g., telerehabilitation and e-PROMs) and a biofeedback layer (e.g., sensors and virtual reality), with dashed arrows indicating the flow of digital information into assessment and monitoring. The framework is interpreted through an implementation science lens (COM-B, RE-AIM, CFIR) and grounded in digital equity and cultural responsiveness.

The main contribution of the DPPF is that it organizes digital technologies according to their clinical role across the care pathway, rather than by device type alone. This is particularly relevant because the current literature remains largely modality-specific. Trials tend to examine VR, telehealth, robotics, exergaming, or wearable systems separately, even though pediatric physiotherapy is rarely delivered in such isolated ways in routine practice [36,37,39,40,44,47,50,54]. By shifting the unit of analysis from the device itself to its clinical function, the framework offers a more integrated and clinically meaningful way of understanding digital pediatric physiotherapy. The overall structure of the DPPF and its organization across the six stages of care are illustrated in Figure 3.

At its center, the framework retains the child and family as the clinical core, with the ICF-CY providing the conceptual anchor for goals and outcomes across body functions, activities, participation, and contextual factors [19,20,21]. This is an important strength because it keeps digital innovation linked to rehabilitation purpose rather than to technology alone. Around this core, the six-stage care pathway provides a clinically structured way to position digital functions across referral and triage, assessment, care planning, intervention delivery, monitoring and review, and transition/long-term follow-up. The framework is not intended to be a rigid sequence, but rather a practical model for incorporating digital tools into pediatric physiotherapy.

Moreover, the framework treats digital functions as hybrid and interdependent functions. Communication technologies, remote assessment tools, sensor-based monitoring, and immersive treatment platforms rarely operate independently in clinical practice. In telerehabilitation, observation, coaching, and follow-up can be provided, whereas in wearables and VR, objective measurement, feedback, repetition, or engagement can be provided [2,39,40,47,50,54,61]. In this framework, these modalities are not viewed as competing options, but rather as complementary parts of a digitally enabled pediatric physiotherapy pathway.

### 4.2. Six Pathway Stages and Integrated Digital Functions

This six-stage pathway helps clarify where the strongest current evidence is and where important gaps remain. The delivery stage is the strongest, with VR, gaming, tele-exercise, and robot-assisted training were used most frequently to deliver direct interventions [36,37,41,43,44,45,46,49,50,56,59]. Many of these interventions reported favorable short-term effects on motor skills, balance, pain, and fitness. Digital rehabilitation literature shows that the most mature applications of digital tools are for supporting direct treatment, home practice, and therapist-guided activities [1,2,3,61].

Several studies suggest that monitoring is an important development area, although evidence is more limited. Digital tools have been shown to support more continuous clinical review than episodic appointments do, as evidenced by measures of treatment fidelity, adherence tracking, step counts, remote follow-up, or repeated activity monitoring [39,40,47,50,54]. A digital monitoring system can help identify when progress stalls, home practice declines, or intervention plans need to be adjusted. The pediatric monitoring strategies that are most effective, reliable, and acceptable in routine practice remain limited in robust pediatric evidence [61,63,64].

The earliest and latest stages of the pathway remain the least studied. At the referral and triage stage, structured e-referrals, digital intake systems, brief parent-completed screeners, and automated prioritization tools may improve transparency and service coordination, but these functions were not directly tested in the included pediatric rehabilitation trials [61,62,63]. Similarly, at the assessment and goal-setting stage, video-based review, asynchronous home videos, and digitally captured patient- or parent-reported outcomes may help therapists observe children in more natural contexts and support shared, ICF-aligned goals, yet the direct pediatric evidence remains relatively sparse outside selected telehealth studies [2,47,61].

The transition and long-term follow-up stage is especially important because it remains one of the largest gaps in the evidence base. In principle, digital goal tracking, e-PROMs, periodic tele-check-ins, activity monitoring, and shared dashboards could help determine whether gains achieved in therapy are sustained in home, school, and community settings [2,61,63]. These functions may be particularly relevant for children whose needs evolve over time or whose participation is influenced by school transitions, developmental change, or shifts in family support. However, the included trials rarely followed children long enough, or with the necessary outcome breadth, to evaluate these pathway functions directly [37,47,53,54]. For this reason, the transition-related elements of the framework should be viewed as forward-looking and hypothesis-generating, rather than as conclusions already established by the current evidence. A more detailed description of the six stages and the proposed digital functions associated with each stage is provided in Table 4.

The use of COM-B, RE-AIM, and CFIR strengthens the interpretive value of the framework by helping explain why interventions that appear promising in trials may still be difficult to implement routinely [22,23,24,25]. These frameworks were not used to replace the ICF-CY; rather, they complement it by addressing behavioral uptake, service-level implementation, and contextual determinants that are not fully captured by a biopsychosocial outcome framework alone [19,22,25].

It is important that children, caregivers, and clinicians have the ability, opportunity, and motivation to participate in digital pediatric physiotherapy from a COM-B perspective [22]. There were recurring practical themes across the included studies, particularly in home-based VR and telehealth delivery [39,47,53,54], including family training, therapist support, treatment fidelity, and adherence. According to these findings, the success of an intervention depends not only on the technology’s capabilities, but also on its integration into daily life.

As a result of RE-AIM, the evidence base remains heavily biased toward effectiveness, with a comparatively limited number of studies reporting reach, adoption, implementation, and maintenance [23,24]. Although most trials report positive results, they often provide limited information regarding who was excluded, what resources were required, whether the intervention was consistently adopted by clinicians, how fidelity was maintained, or whether benefits were sustained beyond the study period [23,24,39,40,53]. It would be therefore more relevant for real-world services for digital pediatric physiotherapy to incorporate implementation outcomes in future trials.

There are several contextual influences that persist across the review and related literature from a CFIR perspective: device complexity, training requirements, bandwidth and connectivity, organizational readiness, workflow integration, and family capacity to use digital care [2,25,61,62,63]. Pediatric physiotherapy involves a wide range of environments, including clinics, homes, schools, and communities. According to the implementation science interpretation, the field does not lack promising digital tools, but rather lacks sufficiently robust evidence on how to embed them sustainably, equitably, and at scale.

### 4.3. Equity and Cultural Responsiveness

In any framework intended to guide digital pediatric physiotherapy, equity and cultural responsiveness are important. Current empirical evidence on these issues is limited. Digital access barriers, language barriers, socioeconomic constraints, and culturally responsive adaptation were not reported in most included studies conducted in relatively well-resourced settings. Therefore, it is difficult to determine whether digital pediatric physiotherapy narrows or widens inequities in the literature.

As a result of this gap, digital participation is not only technical; it is also social and structural. Internet access, device availability, digital literacy, home space, language preferences, and confidence in remote care can all affect who is able to participate and who benefits [2,6,61]. The evidence from telehealth in pediatric rehabilitation, including Indigenous children and remote communities, suggests that digital approaches may improve access for some families while simultaneously introducing new risks if affordability, cultural safety, and family context are not explicitly taken into account when designing services [2,6,61].

Therefore, the equity considerations summarized in Table 5 should not be considered optional enhancements but rather implementation requirements. Implementation science and pediatric telehealth literature support the use of hybrid pathways, low-bandwidth platforms, multilingual materials, caregiver training, universal design, and monitoring reach across subgroups of the population [2,6,22,23,24,25,61]. Rather than addressing equity after digital models have already been deployed, future work should address equity prospectively in trial design, service evaluation, and policy planning.

### 4.4. Practice and Policy Implications

The pragmatic value of this model emerges by directing professionals and healthcare administrators toward purposefully embedding technological capabilities synchronized with treatment phases and rehabilitation targets, deliberately transcending random integration of standalone digital instruments. For physiotherapy specialists, such paradigms strictly mandate selecting resources fundamentally anchored within pediatric clinical objectives, household environments, and systemic demands, deliberately avoiding adoptions motivated purely through mere hardware accessibility. Furthermore, this necessitates acknowledging that modernized platforms maximize therapeutic effectiveness specifically when they amplify professional rapport, diagnostic reasoning, and conventional physiotherapy whenever those remain medically warranted [2,19,20,21,61].

From an operational standpoint, various consequences necessitate thorough deliberation. Primarily, electronic systems require alignment with specifically articulated clinical roles, such as domestic exercise facilitation, distant therapeutic surveillance, guardian instruction, intervention intensity titration, or methodical longitudinal monitoring. Additionally, deployment strategies must integrate assessments regarding household preparedness, including technological hardware accessibility, caregiver assistance availability, and technical platform proficiency [22,39,47,53,54]. Furthermore, physiotherapy organizations should foresee demands involving professional training, technical assistance coordination, confidentiality safeguards, and emergency contingency protocols for situations where virtual administration appears inadequate or medically unsuitable [2,25,61,62,63].

Regarding healthcare policy, this framework emphasizes the requirement for reimbursement structures, infrastructural financing, and oversight protocols. Viable digital pediatric physiotherapy necessitates fiscal models acknowledging not only synchronous telehealth sessions but also blended clinical administration, remote surveillance data analysis, asynchronous professional tasks, and technical assistance essential for home-based therapeutic regimens [2,61,62,63]. Without these financial structures, modernized methodologies risk remaining restricted to experimental environments instead of progressing into scalable clinical delivery systems. Legislative and institutional structures must further prioritize data confidentiality standards, informed consent protocols, software interoperability, hardware maintenance, and cross-disciplinary coordination. Collectively, these elements dictate whether digital health can be securely and efficiently assimilated into pediatric physiotherapy rehabilitation trajectories [2,25,61,62,63].

### 4.5. Strengths and Limitations of the Current Evidence

The compiled evidence exhibits significant methodological robustness. Analyzed investigations utilized randomized, cluster-randomized, crossover, pragmatic, comparative, or non-inferiority experimental designs, involving heterogeneous pediatric populations and digital rehabilitation platforms. Such diversity suggests digital pediatric physiotherapy has advanced beyond isolated technological or disease-specific applications. Furthermore, implementing pragmatic, crossover, cluster, and non-inferiority frameworks across specific trials strengthens the overall methodological breadth of this research domain [40,46,53].

Nevertheless, substantial methodological limitations remain apparent. Participant cohorts frequently appeared small, therapeutic regimens demonstrated considerable diversity regarding intensity and control selection, endpoint quantification exhibited significant heterogeneity, and follow-up durations were generally short-term [36,37,39,47,54]. Engagement-focused outcomes received minimal scrutiny, while deployment metrics, cost-effectiveness analysis, endurance markers, and fairness variables were rarely reported [22,23,24,25]. Accordingly, contemporary scientific documentation offers encouraging but variable results: most definitive regarding acute physiological and functional improvements, yet significantly more tenuous concerning clinical workflow harmonization, durable participation impact, and prolonged programmatic sustainability.

### 4.6. Limitations of the Framework

Multiple intrinsic constraints define this theoretical structure. Primarily, it constitutes a conceptual design developed through systematic synthesis and professional deduction instead of representing a paradigm subjected to prospective validation within operational environments. Furthermore, while the six-phase arrangement offers heuristic utility, actual pediatric rehabilitation pathways frequently demonstrate recursive and intersecting properties, wherein boundaries dividing evaluation, coordination, administration, surveillance, and discharge stages often lack discrete differentiation. Additionally, since the dominant evidence base stems largely from affluent healthcare settings, the model might insufficiently capture challenges more prevalent in resource-constrained regions, including infrastructural deficits, economic barriers, and fluctuating domestic assistance networks. Moreover, specific framework components; particularly electronic triage systems, e-PROM integration, and automated clinical guidance are included based upon their architectural significance to pathway construction rather than existing definitive pediatric physiotherapy experimental data [2,61,62,63]. This framework must therefore be viewed as a mechanism for structuring current literature and guiding forthcoming investigations alongside clinical expansion projects, instead of being considered a finalized deployment manual.

### 4.7. Research Priorities

Future research needs to move beyond proof-of-concept trials and address the questions most relevant to routine care. These include longer follow-up, better reporting of treatment dose, stronger participation-level outcomes, and more systematic measurement of reach, adoption, fidelity, cost, and maintenance [22,23,24,25]. Comparative studies are also needed to determine when digital delivery is best used as a substitute for in-person care, when it is most useful as an adjunct, and when hybrid pathways are most appropriate.

Research on transition, remote monitoring, e-PROMs, and clinical decision support should be prioritized, as these areas are central to the proposed framework but remain weakly represented in the current evidence base [2,61,62,63]. Equity should likewise be built into study design from the outset through reporting of access barriers, culturally responsive adaptation, and subgroup analyses relevant to underserved families [2,6,61]. Finally, large multisite and practice-embedded studies using more harmonized outcomes will be essential if the field is to move from promising digital interventions to sustainable, patient- and family-centered service models.

## 5. Conclusions

In pediatric physiotherapy, digital health interventions appear to improve selected outcomes primarily at the levels of body functions and activities, including motor performance, balance, upper limb function, pain, and fitness, particularly in the short term. However, the evidence remains heterogeneous and limited by short follow-up, variable study quality, and sparse reporting regarding implementation, equity, and long-term effects. Importantly, there remains a substantial knowledge-to-action gap regarding participation-level outcomes, which are less consistently addressed despite their central importance in child functioning and real-world rehabilitation impact. Within this context, the DPPF provides a structured way to organize current evidence and guide future pathway development; however, some components, particularly digital triage, e-PROMs, clinical decision support, and transition-stage functions, should still be viewed as forward-looking rather than firmly established by this review. In order for digital pediatric physiotherapy to become sustainable in routine practice, it will not only need stronger comparative and longitudinal evidence, but also reimbursement reform, hybrid-care support, infrastructure investment, and equity-focused implementation.

## Figures and Tables

**Table 1:** Characteristics of the included studies.

Author (Year)	Condition/Population	Digital Modality (ICF-CY Focus)	Comparator	Design	*n*	Intervention Dose/Duration	Primary Outcome
Cavalcante Neto 2020 [32]	DCD	Wii training (Activity)	Task-specific exercise	RCT	32	8 weeks; 16 sessions; 60 min/session	MABC-2
Ambrosino 2020 [33]	JRA	Exergaming (Participation)	Usual rehab	Pilot RCT	40	4 weeks inpatient multidisciplinary rehabilitation + 8 weeks home Wii-Fit continuation	6-MWT, CHAQ
Kawasaki 2020 [34]	CP	Hip-robot assist (Activity)	Usual gait	Pilot RCT	10	Sham-controlled crossover on two separate days; one RAGT treadmill session and one non-assisted treadmill session	Gait speed and kinematics
Samhan 2020 [35]	Pediatric hand burns	Robot-enhanced exercise (Activity)	Conventional	RCT	33	8 weeks; 3 sessions/week; 60 min traditional hand rehabilitation + additional 20 min robot-enhanced gaming	AROM, grasp
Jha 2021 [36]	Bilateral spastic CP	VR gaming (Activity)	Usual PT	RCT	38	6 weeks; 4 sessions/week; 60 min/session	GMFM-88, COP sway
Arnoni 2021 [37]	CP	Non-immersive VR (Activity)	Usual gait training	RCT	22	8 weeks; 2 sessions/week; 45 min VR sessions. Control: 50 min/session, 2 sessions/week	TUG, 10 m walk
Kamel 2021 [38]	Hand burns	VR + task training (Activity)	Task training alone	RCT	50	8 weeks; 3 sessions/week; 50 min/session	Jebsen-Taylor, COPM
Farr 2021 [39]	CP	Home-based VR (Activity)	Wait-list	Feasibility RCT	30	12 weeks; 3 sessions/week; 30 min/session; fortnightly physiotherapist support in intervention arm	GMPM, adherence
Hemphill 2022 [40]	Mixed pediatric and adults rehab	VR adjunct (Activity)	Standard PT	Pragmatic RCT	41	Single crossover visit; 10 min VR-assisted PT + 10 min PT-alone, separated by 5 min	Session step-count
Ali 2022 [41]	Burn rehab	VR distraction (Body Fx)	Standard dressing PT	RCT	22	8 weeks; 3 sessions/week; 40 min/session	VAS pain
Basha 2022 [42]	Severe burns	Kinect-VR fitness (Body Fx/Activity)	Matched exercise	RCT	40	12 weeks; 3 sessions/week; 50 min/session	VO_2_ peak, PedsQL
Fu 2022 [43]	CP	VR + robot gait (Activity)	Robot gait alone	RCT	60	12 weeks; 4 sessions/week; 50 min/session, including 20 min VR walking; 48 sessions total	GMFM-66, speed
Arnoni 2022 [44]	CP	Active videogame + PT (Body Fx)	PT alone	RCT	23	8 weeks; 2 sessions/week; 50 min active videogame sessions + conventional therapy. Control: 2 sessions/week; 45 min/session	COP sway, GMFM
Shih 2023 [45]	Unilateral CP	Kinect-CIMT (Activity)	Therapist-CIMT	RCT	29	8 weeks; 2 days/week; 2.25 h/day; 36 h total	Melbourne UL-A
Saussez 2023 [46]	Unilateral CP	Semi-immersive VR in HABIT-ILE (Activity)	HABIT-ILE alone	Non-inferiority RCT	40	2-wk HABIT-ILE day camp; 90 h total. Experimental arm: 53 h HABIT-ILE + 37 h REAtouch	AHA score
Su 2023 [47]	ASD	Telehealth fine-motor and social (Participation)	Face-to-face	Cluster RCT	15	8 weeks; 16 sessions; 60–90 min/session; 2 sessions/week	Fidelity, PDMS-2
Cioffi 2023 [48]	Healthy adolescents	BOXVR vs. YouTube boxing (Body Fx)	Active control	Pilot RCT	42	3 weeks; 5 sessions/week; 10 min/session	STAI, Stroop
Mouhamed 2024 [49]	Ataxic CP (children)	Immersive VR (Body Fx/Activity)	Conventional balance exercise	RCT	64	3 months training	Pediatric Balance Scale
Choi 2024 [50]	CP	Wearable exoskeleton (Activity)	Overground gait without robot	RCT	90	6 weeks; 18 sessions; 30 min/session; 3 sessions/week	Gait speed, 6-MWT
Kurt-Aydin 2024 [51]	DMD/BMD	Immersive VR (Body Fx)	Biofeedback training	RCT	25	12 weeks; 2 sessions/week	6-MWT, pain
Gözaçan 2024 [52]	Unilateral CP	Video-ex gaming (Activity)	Conventional PT	RCT	30	8 weeks	JTTHF, proprioception
Davis 2024 [53]	Rural youth obesity	Telehealth lifestyle (Participation)	School-only	Cluster RCT	148	8 months; 25–26 contact hours total; 15 group sessions + 11 individual/homework sessions	BMI-z
Kilic 2024 [54]	Cystic fibrosis	Tele-exercise (Participation)	Center-based	RCT	39	8 weeks; core stabilization 3 days/week; combined group also performed aerobic exercise 3 days/week	VO_2_ peak, irisin
Raine 2024 [55]	Preadolescents (classroom)	VR classroom aerobic bout (Body Fx)	Sedentary control	Crossover	47	Single acute exercise bout/single session	Math accuracy
Daliri 2025 [56]	CP	VR task-oriented UL training (Activity)	Conventional UL therapy	RCT	30	6 weeks; 3 sessions/week; 45 min/session	QUEST, BBT
Abdel Ghafar 2025 [57]	ASD	Immersive VR balance (Body Fx)	Conventional balance exercise	RCT	53	12 weeks; 3 sessions/week; 30 min/session	Sensory Organization Test
Ali 2025 [58]	Burn children	VR vs. whole-body vibration (Body Fx)	WBV	Comparative RCT	40	8 weeks (two successive months); 3 sessions/week	MMT, FIM-Kids
Yenilmez 2026 [59]	Hemiparetic CP	VR adjunct (Activity)	Exercise only	RCT	31	12 weeks; additional VR twice/week	GMFM-66, PBS
Cavalcante Neto 2026 [60]	DCD	Wii training (Body Fx)	Wait-list	RCT	32	8 weeks; 2 sessions/week; 42 min/session	HRV (RMSSD)

6-MWT = 6-Minute Walk Test; AHA = Assisting Hand Assessment; AROM = active range of motion; ASD = autism spectrum disorder; BBT = Box and Block Test; BMI-z = body mass index z score; CHAQ = Childhood Health Assessment Questionnaire; CIMT = constraint-induced movement therapy; COP = center of pressure; COPM = Canadian Occupational Performance Measure; CP = cerebral palsy; DCD = developmental coordination disorder; DMD/BMD = Duchenne and Becker muscular dystrophy; FIM-Kids = Functional Independence Measure for Children; Fx = function; GMFM-66/88 = Gross Motor Function Measure-66/88; GMPM = Gross Motor Performance Measure; HABIT-ILE = Hand-Arm Bimanual Intensive Therapy Including Lower Extremities; HRV = heart rate variability; ICF-CY = International Classification of Functioning, Disability and Health for Children and Youth; JRA = juvenile rheumatoid arthritis; JTTHF = Jebsen–Taylor Test of Hand Function; MABC-2 = Movement Assessment Battery for Children, Second Edition; Melbourne UL-A = Melbourne Assessment of Unilateral Upper-Limb Function; MMT = manual muscle testing; PDMS-2 = Peabody Developmental Motor Scales, Second Edition; PBS = Pediatric Balance Scale; PedsQL = Pediatric Quality of Life Inventory; PT = physiotherapy; QUEST = Quality of Upper Extremity Skills Test; RAGT = robot-assisted gait training; RMSSD = root mean square of successive differences; RCT = randomized controlled trial; STAI = State–Trait Anxiety Inventory; TUG = Timed Up and Go; UL = upper limb; VAS = visual analogue scale; VO_2_ peak = peak oxygen uptake; VR = virtual reality; WBV = whole-body vibration; wk = week; mo = month, n = number.

**Table 2:** Outcome-level GRADE summary for key digital rehabilitation comparisons.

Comparison (Population)	Main Outcome	Included Studies	Trials (n)	Narrative Effect Summary *	Certainty of Evidence	Reasons for Downgrading
VR-based balance interventions vs. conventional physiotherapy (CP)	Balance/postural control (Pediatric Balance Scale, COP sway, Kids-Mini-BESTest)	[36,44,49,59]	4 trials (n = 156)	Most studies favored the digital intervention for short-term balance-related outcomes.	Moderate	Imprecision
Non-immersive VR/active videogames vs. usual physiotherapy or wait-list (CP)	Gross motor function/functional mobility (GMFM-66, GMFM-88, TUG, 10 m walk test, GMPM)	[36,37,39,44,59]	5 trials (n = 144)	Several studies favored the digital intervention, but findings were not fully consistent across gross motor and mobility outcomes.	Low	Risk of bias; imprecision
Robot-assisted or wearable gait interventions vs. comparison gait training (CP)	Gait-related performance (gait speed, gait kinematics, 6-MWT, GMFM-66)	[34,43,50]	3 trials (n = 160)	Findings were mixed: some studies reported improvement in selected motor outcomes, but gait-speed and endurance outcomes were not consistently better.	Low	Risk of bias; inconsistency; imprecision
Digital interventions in pediatric burn rehabilitation	Pain and upper-limb/functional outcomes (VAS pain, AROM, grasp strength, Jebsen-Taylor Hand Function Test, COPM, VO_2_ peak, FIM-Kids)	[35,38,41,42,58]	5 trials (n = 185)	Digital interventions generally favored pain and functional outcomes, but the specific outcomes and intervention types varied across studies.	Low	Risk of bias; inconsistency; indirectness
Upper-limb digital training vs. standard therapy (unilateral/hemiparetic CP)	Upper-limb function (Melbourne UL-A, AHA, JTTHF, QUEST, Box and Block Test)	[45,46,52,56]	4 trials (n = 129)	Most studies favored the digital intervention or showed non-inferiority compared with therapist-led treatment.	Low	Risk of bias; imprecision
Telerehabilitation/tele-exercise vs. face-to-face or usual care	Participation and service-related outcomes (PDMS-2, fidelity, BMI-z, VO_2_ peak, shuttle/exercise capacity)	[47,53,54]	3 trials (n = 202)	Findings were mixed across populations and outcome domains.	Very low	Risk of bias; inconsistency; indirectness; imprecision
Digital exercise interventions vs. active comparators (mixed non-CP pediatric populations)	Exercise capacity/fitness-related outcomes (6-MWT, VO_2_ peak, HRV, pain)	[33,42,51,54]	4 trials (n = 144)	Some studies favored digital interventions for exercise capacity or physiological outcomes, but results were inconsistent across populations and measures.	Very low	Risk of bias; inconsistency; indirectness; imprecision

* Narrative effect summaries were based on the primary outcome specified by each trial, or on the main study outcome when no primary outcome was explicitly stated. “Favored” indicates a statistically significant between-group effect in favor of the digital intervention on that outcome. Where findings differed across outcomes or time points, this is described as mixed. Certainty ratings were applied at the outcome/comparison level, not to the review as a whole. Abbreviations: 6-MWT = 6-Minute Walk Test; AHA = Assisting Hand Assessment; AROM = active range of motion; BMI-z = body mass index z score; COP = center of pressure; COPM = Canadian Occupational Performance Measure; CP = cerebral palsy; FIM-Kids = Functional Independence Measure for Children; GMFM-66 = Gross Motor Function Measure-66; GMFM-88 = Gross Motor Function Measure-88; GMPM = Gross Motor Performance Measure; HRV = heart rate variability; JTTHF = Jebsen–Taylor Hand Function Test; Kids-Mini-BESTest = Kids Mini-Balance Evaluation Systems Test; PDMS-2 = Peabody Developmental Motor Scales, Second Edition; QUEST = Quality of Upper Extremity Skills Test; RCT = randomized controlled trial; TUG = Timed Up and Go; VAS = visual analogue scale; VO_2_ peak = peak oxygen uptake; VR = virtual reality.

**Table 3:** Clinical overview of digital health modalities in pediatric physiotherapy.

Digital Modality	Main Clinical Role in Pediatric Physiotherapy	What the Included Evidence Mainly Supports	Practical Strengths	Main Limitations/Evidence Gaps	Studies
Telerehabilitation/tele-exercise	Remote follow-up, caregiver coaching, home-based training, service delivery across distance	Feasibility and comparable outcomes to face-to-face care in selected populations; mixed effects across participation and fitness-related outcomes	Improves access, reduces travel burden, supports home-context care	Limited hands-on assessment/treatment; dependent on connectivity, digital literacy, and caregiver engagement; limited pediatric pathway-level evidence	[47,53,54]
VR and gaming	Task-oriented motor training, balance training, gait-related practice, upper-limb rehabilitation, pain distraction	Short-term benefit for balance, gross motor function, upper-limb activity, pain, and selected body-function outcomes, especially in CP	High engagement, intensive repetition, immediate feedback, adaptable task practice	Heterogeneous protocols and outcomes; equipment/training needs; possible motion sickness or screen fatigue; limited long-term data	[36,37,41,44,46,49,56,59]
Wearable sensors, robotics, and robot-assisted training	Gait training, movement assistance, objective monitoring, treatment-intensity support	Some benefit for selected motor and gait-related outcomes; promising objective movement quantification	Supports repetitive practice, offers objective data, may increase treatment intensity	Smaller evidence base than VR; cost, setup, and integration burden; inconsistent findings across gait outcomes	[34,35,43,50,52]
e-PROMs	Symptom tracking, participation monitoring, family-reported outcomes, longitudinal follow-up	Not directly evaluated as a stand-alone digital intervention in the included trials	Potential for remote monitoring, pre-visit data capture, longitudinal tracking	Evidence gap in pediatric physiotherapy; limited direct evaluation of feasibility, psychometrics, workflow integration, and accessibility	[38,42,53]
Digital triage and e-referral	Initial screening, referral prioritization, pathway routing, early service access	Not directly evaluated as a stand-alone intervention in the included trials	Potential to streamline pathways and improve coordination	Very limited direct pediatric evidence; unclear accuracy, fairness, and workflow performance	[40,47,53]
CDSS	Support for intervention planning, treatment selection, and protocol consistency	Not directly evaluated as a stand-alone intervention in the included trials	Potential to support standardized decisions and less experienced clinicians	Underdeveloped in pediatric physiotherapy; no direct trial-based evidence in this review; risk of over-reliance and implementation burden	[40,47,53]

CDSS = clinical decision support systems; CP = cerebral palsy; e-PROMs = electronic patient-reported outcome measures; VR = virtual reality.

**Table 4:** Digital Pediatric Physiotherapy Framework: Care Pathway and Digital Functions.

Pathway Stage	Key Digital Functions	Typical Technologies	ICF-CY Focus	What It Looks Like in Practice
Referral and triage	E-referral and digital intake Algorithm-based triage Self-booking and welcome media	– Web referral forms and provider portals – Rule-based triage engines – Short online screeners	Environmental factors (service access); personal factors (resources, preferences); participation (service utilization)	Physician submits structured e-referral; family completes brief online screener; urgent cases auto-flagged and booked earlier.
Initial assessment	Live tele-assessment e-PROMs collection Asynchronous home-video capture	– Secure video platforms – Upload apps (e.g., cloud links) – Digital history templates	Body functions/structures; activities; participation; contextual factors	Therapist observes mobility via video; parents complete e-PROMs; family uploads short clips of typical play at home.
Goal setting and care planning	Interactive goal-setting tools Shared electronic care plans	– Online SMART/GAS apps – Family portals with editable plans	Activities and participation goals; environmental supports/barriers; personal interests and values	Therapist and family set SMART, ICF-CY-aligned goals using an online tool; plan shared with school therapist.
Intervention delivery	Live tele-rehab sessions App-guided home programs VR/exergaming Wearable feedback	– Video coaching platforms – m-Health exercise apps with reminders and videos	Body functions (strength, balance); activities (gait, self-care); participation (play, sport); environmental routines	Weekly tele-coaching with caregiver; app-based home exercises with reminders; clinic-based VR balance games.
Monitoring and review	Scheduled e-PROMs Wearable and activity data feeds Clinician–family dashboards and alerts	– Wrist accelerometers – Integrated progress dashboards	Activities (daily activity, mobility); participation changes; selected body functions	Quarterly accelerometer wear; e-PROMs trends and activity levels reviewed in a brief tele-consult.
Transition and long-term follow-up	Digital transition plans Periodic tele-check-ins Online self-management and peer resources	– Transition-planning templates – Scheduled tele-visits (e.g., 6 and 12 month)	Participation (school, leisure roles); environmental cross-sector supports; personal self-management skills	Shared digital transition plan from early intervention to school; 6–12-month telehealth reviews; access to online self-management resources.

ICF-CY = International Classification of Functioning, Disability and Health for Children and Youth; e-PROMs = Electronic patient-reported outcome measures; GAS = Goal Attainment Scaling; VR = Virtual reality; m-Health = Mobile health.

**Table 5:** Equity and cultural responsiveness considerations in digital pediatric physiotherapy.

Equity/Cultural Issue	Populations Most Affected	Manifestation in Digital Pediatric Physiotherapy	Key Mitigation Strategies	Linked Framework Elements
Digital access and affordability	Low-income; rural/remote; marginalized communities	Families cannot join video visits or use apps/e-PROMs	Device/data support; community access points; low-bandwidth options	ICF-CY: environmental factors; RE-AIM: Reach; CFIR: outer/inner setting (resources)
Low digital literacy	Older caregivers; families with limited prior technology use	Difficulty logging in or navigating platforms; missed sessions	Simple interfaces; plain-language guides; brief training and live tech support	ICF-CY: environmental factors; COM-B: capability; RE-AIM: Reach/Adoption; CFIR: individuals
Language and cultural barriers	Non-dominant language speakers; Indigenous and culturally diverse communities	Poor understanding of instructions; low trust or engagement	Multilingual materials; interpretation; culturally adapted content; co-design with communities	ICF-CY: participation, environmental factors; RE-AIM: Reach/Adoption; CFIR: outer setting, culture
Home environment and safety	Families in overcrowded, unstable, or unsafe housing	No private space; concerns about confidentiality or safety	Flexible timing; clinic/community digital access; privacy and safety screening	ICF-CY: environmental factors; RE-AIM: Reach/Implementation; CFIR: outer/inner setting
Disability-related accessibility barriers	Children and caregivers with sensory, motor, or cognitive impairments	Difficulty seeing, hearing, or handling devices	Universal design; alternative input methods; caregiver assistance; pacing and breaks	ICF-CY: body functions, activities, environmental factors; RE-AIM: Effectiveness; CFIR: intervention

e-PROMs = Electronic patient-reported outcome measures; ICF-CY = International Classification of Functioning, Disability and Health for Children and Youth; RE-AIM = Reach, Effectiveness, Adoption, Implementation, Maintenance; CFIR = Consolidated Framework for Implementation Research.

## Data Availability

Data will be available from the corresponding author upon request.

## Supplementary Materials

The following supporting information can be downloaded at https://www.mdpi.com/article/10.3390/children13040541/s1, Supplementary Figure S1. Traffic-light plot and Risk of Bias evaluation.
