# Supplementary material for: The Digital Pediatric Physiotherapy Framework (DPPF): A Systematic Review of Digital Health Integration in Pediatric Physiotherapy
Source: Children (Basel). 2026 Apr 13;13(4):541. doi: 10.3390/children13040541 (PMC13115162; doi:10.3390/children13040541)
Supplement: Supplementary file 1 [file children-13-00541-s001.zip › children-4185892-supplementary.pdf]

Supplementary Figure S1. Traffic-light plot and Risk of Bias evaluation

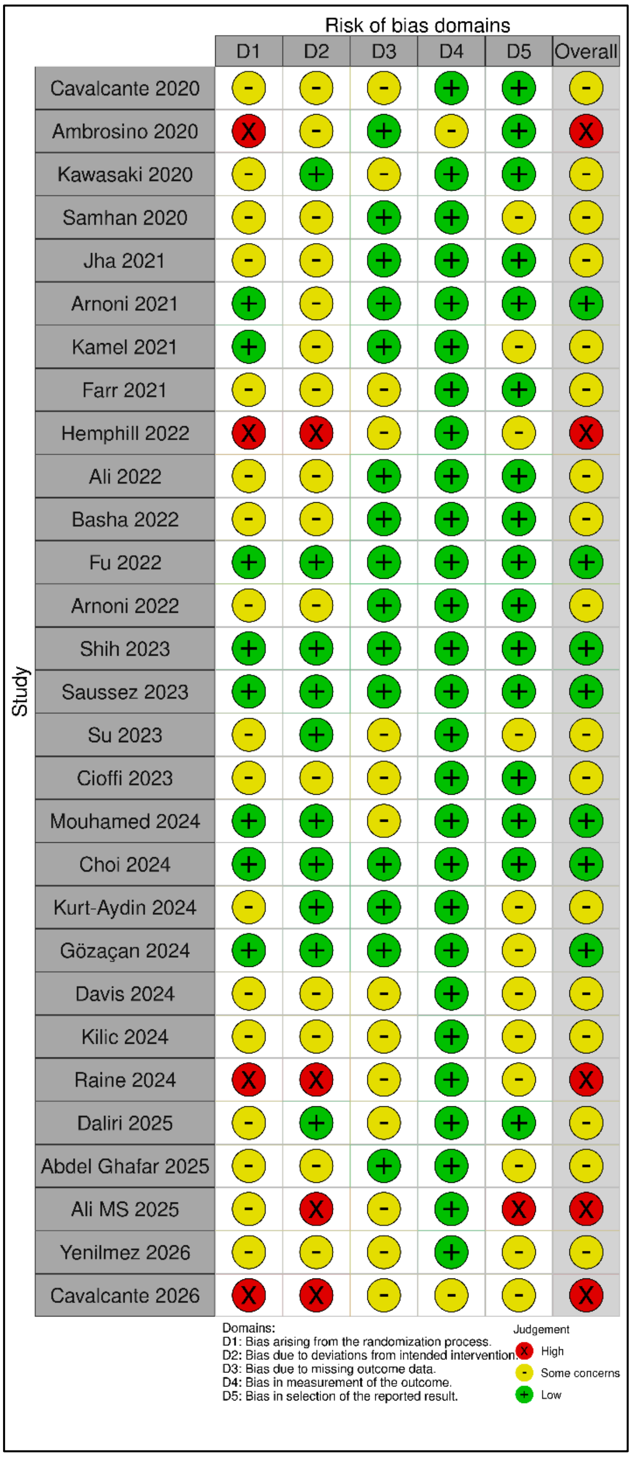

Figure S1. Risk of bias assessment of the included studies using the Cochrane Risk of Bias 2 (RoB 2) tool. The figure presents judgments for each study across five domains: bias arising from the randomization process (D1), bias due to deviations from intended interventions (D2), bias due to missing outcome data (D3), bias in measurement of the outcome (D4), and bias in selection of the reported result (D5), together with the overall risk of bias judgment. Green indicates low risk, yellow indicates some concerns, and red indicates high risk of bias.
